# Imaging Modalities for Diagnosis of Deep Pelvic Endometriosis: Comparison between Trans-Vaginal Sonography, Rectal Endoscopy Sonography and Magnetic Resonance Imaging. A Head-to-Head Meta-Analysis

**DOI:** 10.3390/diagnostics9040225

**Published:** 2019-12-17

**Authors:** Marco Noventa, Marco Scioscia, Michele Schincariol, Francesco Cavallin, Giovanni Pontrelli, Bruna Virgilio, Salvatore Giovanni Vitale, Antonio Simone Laganà, Francesco Dessole, Erich Cosmi, Donato D’Antona, Alessandra Andrisani, Carlo Saccardi, Amerigo Vitagliano, Guido Ambrosini

**Affiliations:** 1 Department of Women and Children’s Health, University of Padua, Padua, 35100, Italy; cosmi@gmail.com (E.C.); dantona@gmail.com (D.D.); andrisani@gmail.com (A.A.); saccardi@gmail.com (C.S.); vitagliano@gmail.com (A.V.); ambrosini@gmail.com (G.A.); 2 Department of Obstetrics and Gynecology, Policlinico Hospital of Abano Terme, Piazza Cristoforo Colombo, 1, 35031 Abano Terme, Italy; marcoscioscia@gmail.com (M.S.); pontrelli@gmail.com (G.P.); virgilio@gmail.com (B.V.); 3 Medizinische Klinik I, Klinikum Furth, Jakob-Henle-Straße 1, 90766 Fürth, Germany; schinchiariol@gmail.com; 4 Independent statistician, Solagna, 36020, Vicenza, Italy; cava@gmail.com; 5 Department of General Surgery and Medical Surgical Specialties, University of Catania, 2, 95124 Catania, Italy; vitale@gmail.com; 6 Department of Obstetrics and Gynecology, Filippo Del Ponte Hospital, University of Insubria, 2, 21100 Varese, Italy; lagana@gmail.com; 7 Gynecologic and Obstetric Clinic, Department of Surgical, Microsurgical and Medical Sciences, University of Sassari, 21, 07100 Sassari, Italy; dessole@gmail.com

**Keywords:** endometriosis, ultrasonography, magnetic resonance imaging, diagnostic tests, sensitivity and specificity, early diagnosis, pelvis

## Abstract

Objectives: A meta-analysis, with a head-to-head approach, was carried out to compare the three most common techniques for a deep pelvic endometriosis (DPE) diagnosis. We focused on: transvaginal-sonography (TVS), magnetic-resonance imaging (MRI), and rectal-endoscopy-sonography (RES). Methods: Electronic databases were searched from their inception until December 2018. All prospective and well-defined retrospective studies carried out in tertiary referral centers were considered. This review was reported following the Preferred Reporting Items for Systematic Reviews and Meta-Analyses (PRISMA) and Synthesizing Evidence from Diagnostic Accuracy Tests (SEDATE) guidelines. We considered only papers in which at least two imaging modalities were compared in the same set of patients (head-to-head approach). Meta-analysis of diagnostic test accuracy (DTA) was performed separately for each location of interest. Bivariate or univariate approach has been applied when appropriate. We analyze the DTA of TVS vs. MRI, TVS vs. RES, and MRI vs. RES. Results: Our meta-analysis (17 studies included) showed high-to-moderate DTA of TVS for all endometriosis locations (apart from recto-vaginal septum (RVS)) that were not statistically different from MRI and RES for those localized in the posterior compartment. RES results were more accurate than MRI for RS lesions but less accurate than TVS for other pelvic locations, except for RVS. Conclusions: All approaches provide good accuracy with specific strong points. Ultrasonography demonstrated a diagnostic accuracy not inferior to MRI and RES; therefore, it must be considered the primary approach for DPE diagnosis. MRI has to be considered as a valuable approach in settings where highly skilled sonographers are not available. Keypoints: (1) We confirmed the non-inferiority of TVS compared to MRI and RES for the diagnosis of specific pelvic anatomic location of endometriosis lesions. (2) Ultrasonography could be considered the primary approach for DPE diagnosis (less invasive than RES and less expensive than MRI). (3) MRI has to be considered as a valuable approach in settings where skilled sonographers are not available.

## 1. Introduction

The prevalence of deep pelvic endometriosis (DPE) is estimated in 15–30% of all women affected by the disease; it is a source of chronic pelvic pain, infertility, and reduced quality of life [1,2,3,4]. Different imaging modalities have been proposed for its early diagnosis with contrasting results [1,2,3,4]. The most studied techniques are trans-vaginal sonography (TVS), magnetic resonance imaging (MRI), and rectal-endoscopy sonography (RES) [1,2,5].

TVS is proposed as a first line technique, however its diagnostic accuracy varies considerably both for the same and for different DPE locations [1,2,6,7,8,9]. RES demonstrated a good diagnostic accuracy especially for recto-sigmoid (RS), recto-vaginal septum (RVS), and vaginal-wall and vaginal-fornix (VW & VF), but it may be considered as a second-line approach due to its invasiveness [1]. Finally, two recent meta-analyses suggested a good diagnostic accuracy of MRI for all DPE locations, but it seems only slightly superior compared to TVS [6,7,9,10].

Taking these data together and considering the fundamental role of an early diagnosis of DPE, we must definitively understand which imaging modality (between TVS, RES, and MRI) can guarantee the greatest diagnostic accuracy according to different suspected DPE locations to give the reader clear suggestions on which method to use according to the suspected localization and diagnostic setting.

## 2. Methods

### 2.1. Study Design and Protocol Registration

This is a meta-analysis of only head-to-head studies comparing the diagnostic accuracy of three different imaging techniques for the diagnosis of DPE. We analyzed data regarding the following comparisons: (i) TVS versus MRI; (ii) TVS versus RES; and (iii) MRI versus RES. The DPE locations reported and analyzed were: utero-sacral ligaments (USLs), vaginal-wall and vaginal fornix (VW & VF), bladder (BL), recto-sigmoid (RS), pouch of Douglas (PoD), and recto-vaginal septum (RVS).

This review was reported following the Preferred Reporting Items for Systematic Reviews and Meta-Analyses (PRISMA) and the Synthesizing Evidence from Diagnostic Accuracy Tests (SEDATE) guidelines [11,12]. See PRISMA in Figure S1. PROSPERO registration number CRD42019116065.

### 2.2. Eligibility Criteria

We included in this meta-analysis prospective or retrospective cohort studies including patients who underwent at least two diagnostic techniques between TVS, MRI, and RES for the diagnosis of DPE. All included studies were required to describe the number of patients affected and the anatomical location of the lesions. We considered eligible for our review only papers in which the decision for surgery (performed in all patients included in the study) was based on clinical symptoms.

The inclusion criteria applied in this meta-analysis were the following:

- Type of study: No restriction.
- Participants: Patients with clinical suspicion of DPE based on clinical complaints and/or physical examination.
- Index test: TVS, MRI, and RES were considered as index test.
- Reference test: Surgical procedure and histological examination were the reference standard of this meta-analysis according to literature [1].
- Outcome: According to our sections, we analyzed the diagnostic accuracy of each technique compared to one another (one-to-one comparison: TVS vs. MRI; TVS vs. RES, and MRI vs. RES) for each DPE location. The DPE locations reported and analyzed were: USLs, VW & VF, BL, RS, PoD and RVS.
- True positive (TP), false positive (FP), true negative (TN), and false negative (FN) were retrieved or calculated if necessary.
- Setting: Tertiary center hospitals
- Data required for extraction: Information sufficient to produce a 2 × 2 table for calculation of sensitivity and specificity.
- Language: No language restrictions were applied.

### 2.3. Information Sources and Search Strategies

Electronic databases (Medline, Scopus, Embase, Sciencedirect, Cochrane library, Clinicaltrials.gov, Cochrane Central Register of Controlled Trials, EU Clinical Trials Register and World Health Organization International Clinical Trials Registry Platform) were searched from their inception until December 2018.

According to the sections analyzed, we performed three independent searches. In the first section, key search terms were the following text words: transvaginal ultrasound/sonography/ultrasonography [Mesh] AND deep pelvic endometriosis OR endometriosis OR deep infiltrating endometriosis OR utero-sacral ligaments endometriosis OR vaginal/vaginal fornix endometriosis OR bladder endometriosis OR colon/recto-sigmoid endometriosis OR pouch of Douglas endometriosis OR recto-vaginal septum endometriosis. For the second and third sections we used as key search terms rectal sonography/ultrasound/ultrasonography [Mesh] and magnetic resonance imaging [Mesh], respectively adding the following terms as above.

### 2.4. Study Selection and Data Extraction

Titles and abstracts were independently screened by two authors (A.V.; M.N.). The same authors independently assessed studies for inclusion and extracted data about study features, populations, type of intervention, and outcomes. A manual search of references of included studies was also performed to avoid missing relevant data. The results were compared and any disagreement was resolved by consensus.

Specifically, for our outcomes we collected data about: prevalence of disease, sensitivity, specificity, positive predictive value (PPV), negative predictive value (NPV), absolute number of true positive (TP), false positive (FP), true negative (TN), and false negative (FN). In the case of missing data about TP, FP, TN and FN, we calculated them starting from prevalence, sensitivity, and specificity.

We excluded from the analysis narrative or systematic reviews, case reports/case series, and conference abstracts.

### 2.5. Risk of Bias in Individual Studies

According to the recommendation provided by The Cochrane Collaboration [13], the quality assessment was conducted by the tool provided by the Quality Assessment of Diagnostic Accuracy Studies-2 (QUADAS-2) [14].

The QUADAS-2 format includes four domains: (1) patient selection, (2) index test, (3) reference standard, and (4) flow and timing. For each domain, the risk of bias and concerns about applicability (the latter not applying to the domain of flow and timing) were analyzed and rated as low, high or unclear risk (Table 1). Three authors (A.V., M.N. and F.C.) independently evaluated the methodological quality using a standard form with quality assessment criteria and a flow diagram. Any disagreement was resolved by consensus.

### 2.6. Statistical Analysis

Statistical analysis was performed using the package “mada” [32] of R 3.3.2 (R Foundation for Statistical Computing, Vienna, Austria) [33]. A *p*-value less than 0.05 was considered statistically significant.

This study included only head-to-head studies, thus diagnostic test accuracy (DTA) was evaluated according to each comparison of detection methods (TVS vs. RMN; TVS vs. RES and RMN vs. RES). In addition, meta-analysis of DTA was performed separately for each location of interest (RS colon/rectosigmoid; recto vaginal septum; USLs; vaginal and posterior vaginal fornix; pouch of Douglas; bladder).

Since the sensitivity and the specificity of a diagnostic test are interrelated, the bivariate approach to the meta-analysis of DTA [34] was preferred to the univariate approach for locations with 5 or more studies [35]. The bivariate approach included the estimation of a bivariate normal model for the logit-transformed pairs of sensitivities and false positive rates (FPR) [35]. This model was estimated as a linear mixed model with random effects, according to Arends et al. [36]. DTA was compared within each pair of detection methods with HSROC (Hierarchical summary receiver operating characteristic) curves and meta-regression [37].

Heterogeneity is to be expected in meta-analyses of DTA [13]. While univariate measures of heterogeneity as the I2 statistic are not appropriate in meta-analyses of DTA, the magnitude of observed heterogeneity can be evaluated graphically by the scatter of points and the prediction ellipse [13].

If the number of studies evaluating a specific location was less than 5, the univariate approach to the meta-analysis of DTA was adopted. Since pooling sensitivities or specificities can be misleading [13], univariate measures of accuracy like the diagnostic odds ratio (DOR), the positive likelihood ratio (LR+), and the negative likelihood ratio (LR−) were pooled with descriptive purpose.

During the study design, we planned to assess the risk of publication bias using Deeks’ funnel plot [38]. However, the limited number of included studies for each combination of location and pair of detection methods did not allow any meaningful investigation of publication bias [13].

## 3. Results

### 3.1. Study Selection

**Figure 1:**
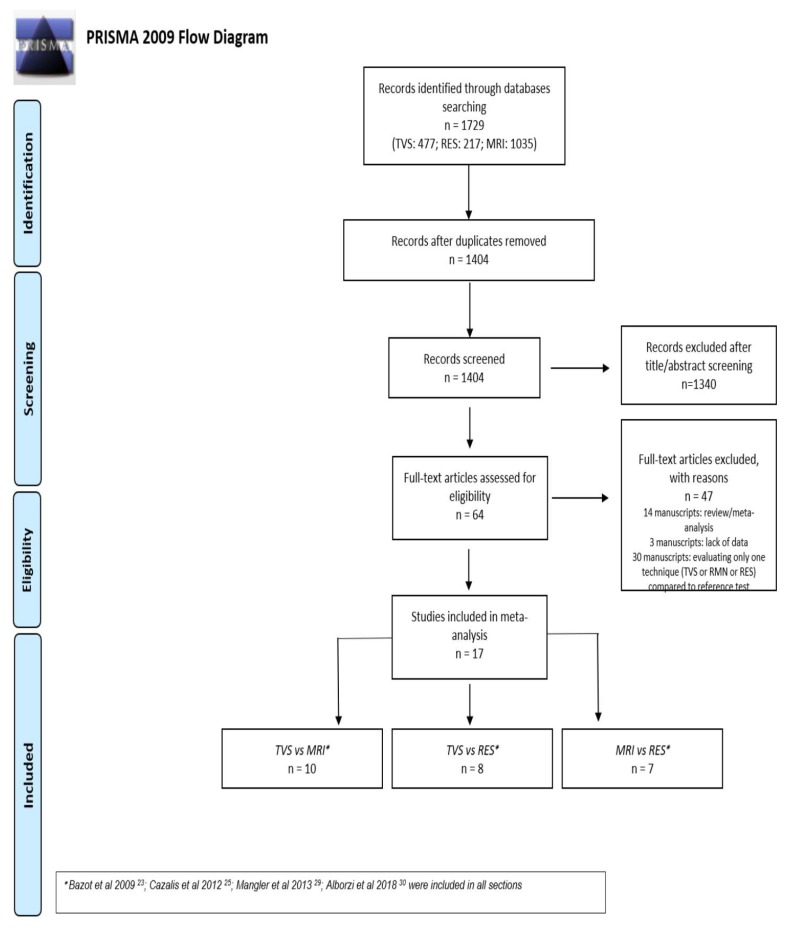
Flow diagram of included studies.

The electronic searches provided a total of 1729 citations but after the removal of 327 duplicate records, 1404 citations remained. Of these, 1340 records were excluded after title/abstract screening (not relevant to the review). We examined the full text of 64 remaining manuscripts and, of these, we excluded 47 papers (three papers due to lack of data concerning diagnostic performance, 14 papers because they were review/meta-analysis, and finally 30 papers because the design was not head-to-head and they reported diagnostic accuracy of only one technique without comparison with at least one other). Finally, 17 manuscripts were included in the meta-analysis. Ten manuscripts were included in the first section of TVS versus MRI [17,21,23,24,25,26,27,28,30,31]; eight manuscripts were included in the second section of TVS versus RES [15,18,20,21,22,24,28,30] and finally seven manuscripts were included in the third section of MRI versus RES [16,19,21,24,28,29,30]. Four papers were included in all three sections [21,24,28,30]. A flowchart summarizing literature identification and selection is given in Figure 1.

### 3.2. Diagnostic Performance of TVS Versus MRI

In this section, we included a total of 10 papers [17,21,23,24,25,26,27,28,30,31]. General features including manuscripts, patients, index tests, and reference standards are reported in detail in Table 2.

**Figure 2:**
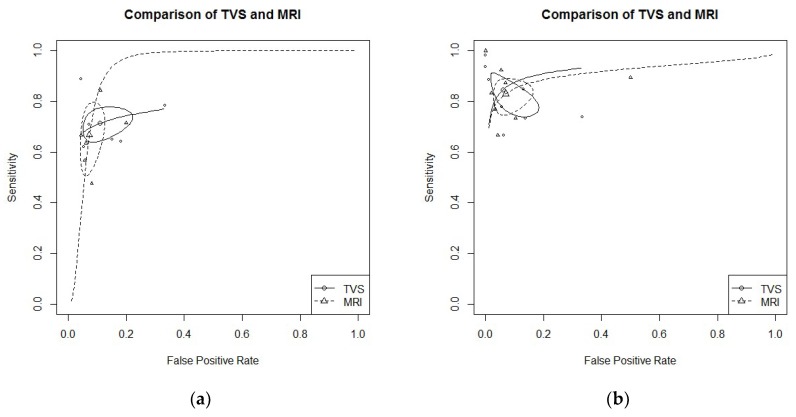

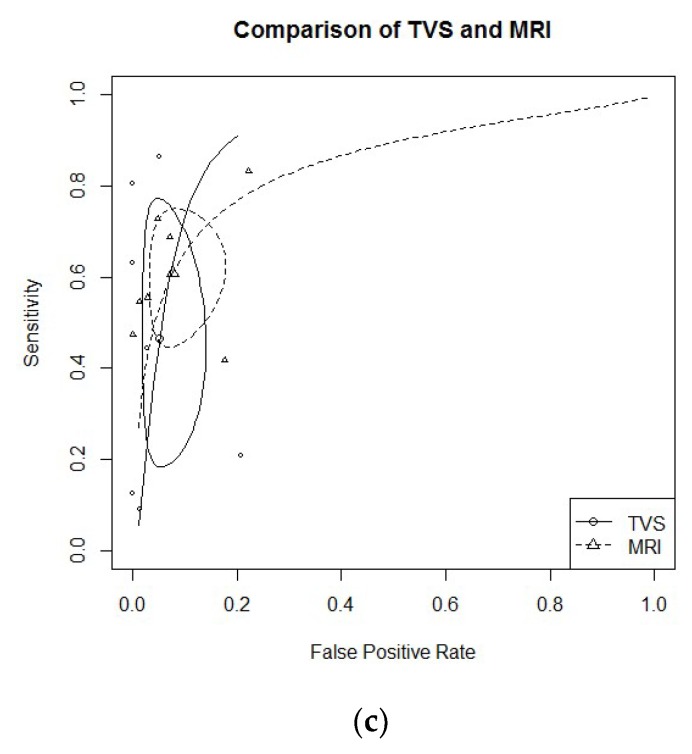
(**a**) TVS vs. MRI for utero-sacral ligaments (USLs) endometriosis detection; bivariate model (HSROC). (**b**) TVS vs. MRI for recto-sigmoid (RS) endometriosis detection; bivariate model (HSROC). (**c**) TVS vs. MRI for recto-vaginal septum (RVS) endometriosis detection; bivariate model (HSROC).

- USLs endometriosis: Six studies evaluated DTA of TVS and MRI for the detection of USLs endometriosis [21,24,25,26,27,30]. Pooled sensitivity and FPR were 0.71 (95% CI 0.65–0.77) and 0.11 (95% CI 0.06–0.19) for TVS, and 0.67 (95% CI 0.54–0.77) and 0.07 (95% CI 0.05–0.11) for MRI (HSROC in Figure 2a). Heterogeneity can be evaluated by predicted ellipses in Figure 2a. Bivariate meta-regression indicated similar accuracy for TVS and MRI (*p* = 0.65).
- RS endometriosis: Eight studies evaluated DTA of TVS and MRI for RS endometriosis detection [17,21,23,24,26,27,30,31]. Pooled sensitivity and FPR were 0.85 (95% CI 0.76–0.90) and 0.06 (95% CI 0.02–0.15) for TVS, and 0.83 (95% CI 0.76–0.88) and 0.07 (95% CI 0.03–0.14) for MRI (HSROC in Figure 2b). Heterogeneity can be evaluated by predicted ellipses in Figure 2b. Bivariate meta-regression indicated similar accuracy for TVS and MRI (*p* = 0.96).
  RVS endometriosis: Seven studies evaluated DTA of TVS and MRI for RVS endometriosis detection [21,24,25,26,27,28,30]. Pooled sensitivity and FPR were 0.47 (95% CI 0.23–0.72) and 0.05 (95% CI 0.02–0.12) for TVS, and 0.61 (95% CI 0.48–0.72) and 0.08 (95% CI 0.04–0.15) for MRI (HSROC in Figure 2c). Heterogeneity can be evaluated by predicted ellipses in Figure 2c. Bivariate meta-regression indicated similar accuracy for TVS and MRI (*p* = 0.47).
  Other localization of endometriosis (univariate approach, TVS vs. MRI):
- Four studies investigated DTA for VW & VF endometriosis detection [21,25,26,27]. By univariate approach, we found a pooled logarithm of DOR of 4.05 (95% CI 2.15–5.96) and 3.28 (95% CI 2.48–4.09) for TVS and MRI respectively.
- Four studies investigated DTA for BL endometriosis detection [24,27,30,31]. By univariate approach, we found a pooled logarithm of DOR of 4.39 (95% CI 2.03–6.74) and 3.09 (95% CI 1.74–6.07) for TVS and MRI, respectively.
- Two studies investigated DTA for PoD endometriosis detection [17,25]. By univariate approach, we found a pooled logarithm of DOR of 5.38 (95% CI 2.27–8.48) and 3.52 (95% CI 0.24–7.29) for TVS and MRI, respectively.

Data of DTA of TVS and MRI by univariate approach (with 95% confidence intervals) are reported in detail in Table S1.

### 3.3. Diagnostic Performance of TVS Versus RES

In this section we included a total of eight papers [15,18,20,21,22,24,28,30]. General features on included manuscripts, patients, index test, and reference standard are reported in detail in Table 3.

**Figure 3:**
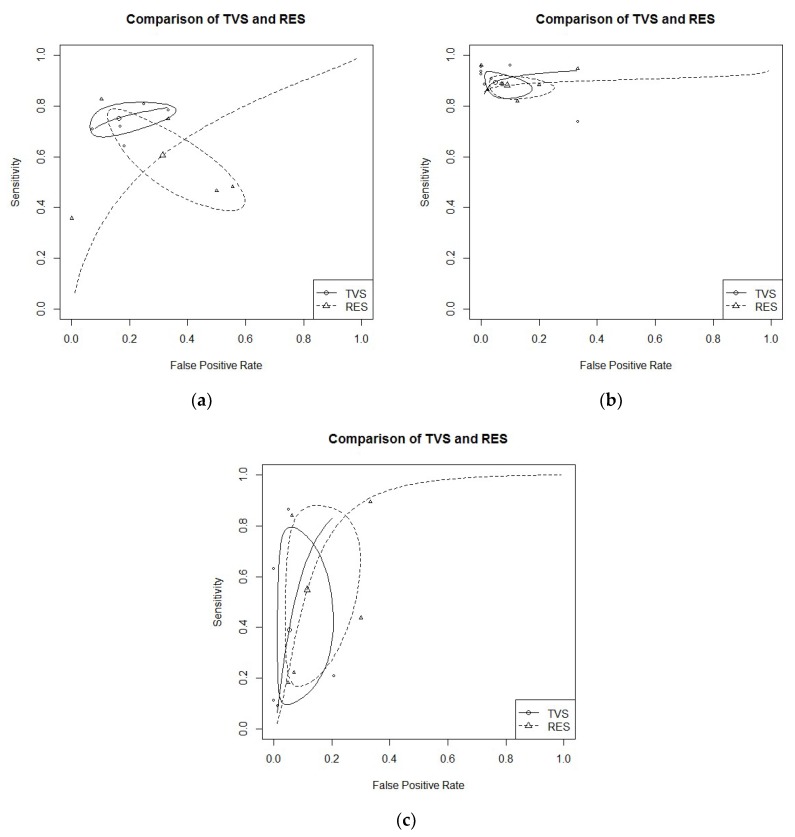
(**a**) TVS vs. RES for USLs endometriosis detection; bivariate model (HSROC). (**b**) TVS vs. RES for RS endometriosis detection; bivariate model (HSROC). (**c**) TVS vs. RES for RVS endometriosis detection; bivariate model (HSROC).

- USLs endometriosis: Five studies evaluated DTA of TVS and RES for USLs endometriosis detection [18,21,22,24,30]. Pooled sensitivity and FPR were 0.75 (95% CI 0.69–0.70) and 0.16 (95% CI 0.08–0.31) for TVS, and 0.61 (95% CI 0.43–0.76) and 0.31 (95% CI 0.15–0.54) for RES (HSROC in Figure 3b). Heterogeneity can be evaluated by predicted ellipses in Figure 3a. Bivariate meta-regression indicated similar accuracy for TVS and RES (*p* = 0.29).
- RS endometriosis: Seven studies evaluated DTA of TVS and RES for RS endometriosis detection [15,18,20,21,22,24,30]. Pooled sensitivity and FPR were 0.89 (95% CI 0.84–0.93) and 0.05 (95% CI 0.02–0.14) for TVS, and 0.88 (95% CI 0.84–0.91) and 0.09 (95% CI 0.04–0.21) for RES (HSROC in Figure 3a). Heterogeneity can be evaluated by predicted ellipses in Figure 3b. Bivariate meta-regression indicated similar accuracy for TVS and RES (*p* = 0.68).
  RVS endometriosis: Five studies evaluated DTA of TVS and RES for RVS endometriosis detection [21,22,24,28,30]. Pooled sensitivity and FPR were 0.39 (95% CI 0.13–0.73) and 0.05 (95% CI 0.02–0.16) for TVS, and 0.55 (95% CI 0.22–0.84) and 0.11 (95% CI 0.05–0.25) for RES (HSROC in Figure 3c). Heterogeneity can be evaluated by predicted ellipses in Figure 3c. Bivariate meta-regression indicated similar accuracy for TVS and RES (*p* = 0.40).
  Other localization of endometriosis (univariate approach, TVS vs. RES):
- Three studies evaluated DTA for VW & VF endometriosis detection [18,21,22]. By univariate approach, we found a pooled logarithm of DOR of 2.88 (95% CI 1.95–3.81) and 1.95 (95% CI 0.42–3.49) for TVS and RES, respectively.
- Two studies evaluated DTA for BL endometriosis detection [24,30]. By univariate approach, we found a pooled logarithm of DOR of 4.94 (95% CI 0.12–10.02) and 3.13 (0.56–5.70) for TVS and RES, respectively.
- Only one study DTA of TVS and RES for PoD endometriosis [21]

Data of DTA of TVS and RES by univariate approach (with 95% confidence intervals) are reported in detail in Table S2.

### 3.4. Diagnostic Performance of MRI Versus RES

In this section we included a total of seven papers [16,19,21,24,28,29,30]. General features on included manuscripts, patients, index test, and reference standard are reported in detail in Table 4.

**Figure 4:**
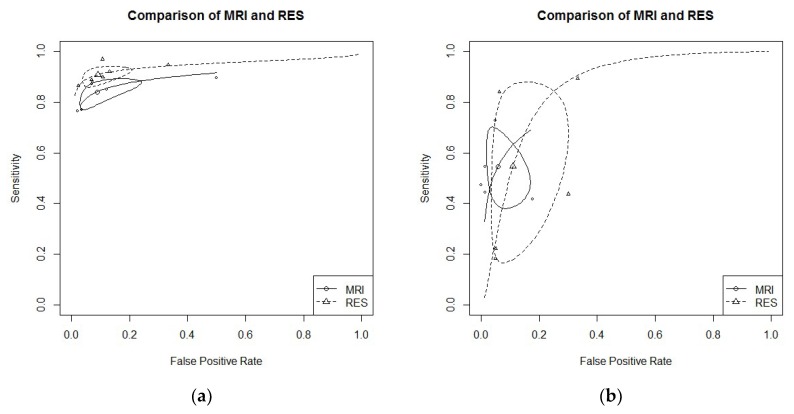
(**a**) MRI vs. RES for RS endometriosis detection; bivariate model (HSROC). (**b**) MRI vs. RES for RVS endometriosis detection; bivariate model (HSROC).

- RS endometriosis: six studies evaluated DTA of MRI and RES for RS endometriosis detection [16,19,21,24,29,30]. Pooled sensitivity and FPR were 0.84 (95% CI 0.79–0.88) and 0.09 (95% CI 0.04–0.20) for MRI, and 0.91 (95% CI 0.87–0.94) and 0.13 (95% CI 0.04–0.18) for RES (HSROC in Figure 4a). Heterogeneity can be evaluated by predicted ellipses in Figure 4a. Bivariate meta-regression indicated different accuracy for MRI and RES (*p* = 0.03). In particular, RES offered a better sensitivity than MRI (*p* = 0.02), while the false positive rate was similar between the two methods (*p* = 0.92).
- RVS endometriosis: five studies evaluated DTA of MRI and RES for RVS endometriosis detection [19,21,24,28,30]. Pooled sensitivity and FPR were 0.55 (95% CI 0.41–0.67) and 0.06 (95% CI 0.02–0.14) for MRI, and 0.55 (95% CI 0.22–0.84) and 0.11 (95% CI 0.05–0.25) for RES (HSROC in Figure 4b). Heterogeneity can be evaluated by predicted ellipses in Figure 4b. Bivariate meta-regression indicated similar accuracy for MRI and RES (*p* = 0.43).
  Other localization of endometriosis (univariate approach, MRI vs. RES):
- Four studies evaluated DTA for USLs endometriosis detection [19,21,24,30]. By univariate approach, we found a pooled logarithm of DOR of 3.28 (95% CI 2.66–3.90) and 1.39 (9% CI 1.19–3.98) for MRI and RES, respectively.
- Two studies evaluated DTA for VW & VF endometriosis detection [19,21]. By univariate approach, we found a pooled logarithm of DOR of 3.08 (95% CI 2.27–3.89) and 2.44 (95% CI 0.27–4.61) for MRI and RES, respectively.
- Two studies evaluated DTA of MRI and RES for BL endometriosis detection [24,30]. By univariate approach, we found a pooled logarithm of DOR of 4.29 (95% CI 1.75–10.35) and 3.13 (95% CI 0.56–5.70) for MRI and RES, respectively.
- No study reported data for PoD endometriosis detection.

Data of DTA of TVS and RES by univariate approach are reported in detail in Table S3.

### 3.5. Risk of Bias Within Studies

Regarding the domain “patient selection,” the overall quality was high, with only one paper judged at high risk of bias [17] and six papers at unclear risk [16,18,22,24,26,29,31]. Regarding the domain “index test,” five papers were judged at unclear risk because it is not clearly reported if operators performing the imaging procedures were blind to the results [17,23,25,26,29]. One paper was judged at high risk because the same operator performed different procedures [30]. However, all papers described the procedures adequately. Regarding the domain “reference standard,” we decided to define all manuscripts at unclear risk of bias because even if all papers used an adequate and accepted reference to define the pathology (surgery and/or histology), they did not specify if the results of the reference standard were interpreted without knowledge of the results of the index test. Finally, concerning the domain “flow and timing,” almost all papers, except for five [17,18,24,27,28], did not report the time interval between the index and reference test.

### 3.6. Applicability Concerns

Regarding the domain “patient selection,” the quality of manuscripts was high with only one paper judged at unclear risk [17] by including patients who did not match the review question. Regarding the applicability of “index test,” all papers except for two of unclear risk [17,28], were judged of good quality (low risk) because they adequately described the methodology of the index test. Finally, considering the applicability of “reference standard,” all papers used surgery or histology, however, seven papers did adequately specify the reference standard used (surgery and/or histology alone or combined) and so were judged to be an unclear risk [15,17,20,25,26,27,28].

Graphical representation of the QUADAS-2 tool is reported in Table 1.

## 4. Discussion

### 4.1. Synthesis of the Results

We confirm the high-to-moderate diagnostic accuracy of TVS for all DPE locations that were not inferior to MRI for USLs, RVS, RS endometriosis and to RES for RS endometriosis. Certainly, its good accuracy is strictly dependent on the setting; in the case of suspected DPE (with clinical guidance), TVS should be performed in a setting dedicated to the diagnosis and care of endometriosis only by highly skilled and trained sonographers [1,2,39,40].

RES showed good diagnostic accuracy for the posterior compartment, especially for RS endometriosis. Its sensitivity results were comparable to TVS and even better compared to MRI. Data from other locations seemed unexciting, but we cannot extrapolate definitive conclusions due to the low number of studies. Certainly, the acceptance of this technique is inferior to both TVS and MRI (even if there are no studies aimed at evaluating this issue) and so it must be applied only as a second-line procedure in a dedicated setting when RS endometriosis is suspected [1].

Finally, MRI showed a good-to-moderate diagnostic accuracy for all sites, not inferior to TVS for USLs and RVS endometriosis and only slightly inferior to RES for RS endometriosis (with sensitivity but not FPR).

### 4.2. Interpretation of the Results

An accurate early diagnosis of DPE is crucial to make a clinical decision and to ensure an adequate treatment in relation to a patient’s symptoms (pain and infertility) and quality of life. Many efforts have been made to search for the ideal diagnostic technique that could ensure the highest accuracy [1]. Unfortunately, many years are often necessary to get to a definitive diagnosis of DPE [3]. This has a multifactorial origin: firstly, DPE is characterized by variable and unspecific symptoms that can mislead general practitioners or non-specialized gynecologists and can result in a delay of diagnosis [1,3]. Secondly, although TVS is a widely used tool in common gynecologic practice, non-expert operators without a specific training in DPE recognition can increase this delay [29].

Of all diagnostic tools for DPE diagnosis, the most studied are TVS and related modified techniques, MRI and RES [1,2,5]. TVS has been indicated as the first-line imaging technique due to its low costs and invasiveness. Differently, according to the European society of urogenital radiology, MRI must be considered a second-line technique [2]. Advantages of MRI are related to the possibility of evaluating the extent of endometriotic lesions in the upper part of the abdomen (localizations outside the pelvic cavity) and the presence of dense adhesions [10]. Disadvantages are related to the cost and invasiveness of the procedure and the fact that it is more time-consuming compared to an ultrasound scan (this last technique is generally immediately available in all gynecologic settings) [1,2].

### 4.3. Comparison to Other Meta-Analysis

Recently, Guerriero et al. published a meta-analysis where they compared by head-to-head approach only TVS and MRI. Different than this study, they applied a bivariate approach when at least four papers described each DPE site [8]. Even if our results are quite similar, we decided to apply more strict criteria to give stronger evidence, performing bivariate analysis only when five or more papers described each DPE site. Moreover, we included in the final analysis four more well conduced observational prospective studies [25,28,30,31] that increased our evidence even further. Finally, even more importantly, we included in the analysis not only one comparison (TVS vs. MRI), but also two more comparisons (TVS vs. RES and MRI vs. RES).

All other meta-analyses described only the diagnostic performance of TVS and modified techniques like MRI, giving estimated pooled sensitivity and specificity without comparing the results to other diagnostic procedures [1,6,7,8,9,10]. Moreover, this is the first meta-analysis that included the diagnostic accuracy of RES and compared the head-to-head approach on all of the most studied imaging modalities (TVS, MRI, and RES) for DPE diagnosis.

### 4.4. Strengths and Limitations

First, the analysis included some retrospective studies because of the paucity of prospective manuscripts evaluating TVS versus RES and MRI versus RES. However, only retrospective studies of good quality were included in the analysis. Second, the recommended procedure for DTA meta-analysis (the bivariate approach) [13] was not reasonably applicable in some locations due to the low number of included studies. New prospective studies with adequate sample sizes are mandatory to confirm our evidence.

Despite these limitations, this is the first meta-analysis that analyzed the diagnostic accuracy of TVS, MRI, and RES for each DPE location in patients who received at least two procedures (with a head-to-head approach). This selection allowed us not only to describe the DTA of each method, but also to compare the diagnostic power of each technique.

## 5. Conclusions

All approaches provide good accuracy with specific strong points. We confirmed the non-inferiority of TVS compared to MRI for the diagnosis of USLs, RVS, and RS endometriosis and to RES for the diagnosis of RS endometriosis. Diagnostic accuracy of RES seemed only slightly better compared to MRI for RS endometriosis. As for all the other localizations, we cannot give specific recommendations; more studies are necessary to reach further conclusions. Ultrasonography must be considered as the primary approach for DPE diagnosis (less invasive than RES and less expensive than MRI). MRI has to be considered as a valuable approach in settings where highly skilled sonographers are not available.

## Figures and Tables

**Table 1:** Risk of bias in individual studies evaluated through the Quality Assessment of Diagnostic Accuracy Studies-2 (QUADAS-2) tool.

Study	Risk of Bias				Applicability Concerns		
	Patient Selection	Index Test	Reference Standard	Flow and Timing	Patient Selection	Index Test	Reference Standard
Bazot et al. 2003 [15]	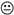	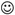	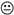	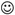	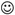	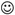	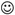
Chapron et al. 2004 [16]	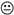	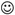	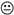	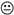	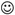	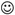	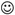
Abrao et al. 2007 [17]	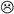	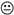	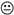	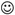	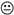	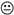	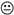
Bazot et al. 2007 a [18]	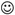	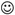	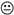	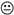	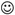	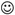	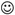
Bazot et al. 2007 b [19]	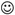	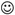	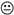	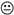	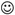	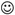	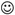
Piketty et al. 2008 [20]	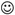	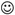	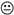	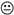	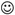	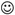	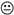
Bazot et al. 2009 [21]	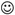	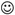	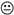	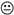	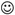	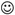	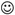
Bergamini et al. 2010 [22]	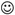	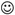	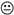	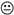	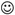	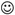	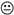
Saba et al. 2012 [23]	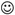	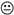	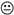	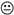	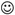	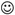	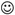
Cazalis et al. 2012 [24]	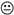	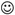	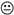	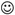	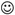	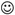	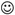
Fiaschetti et al. 2012 [25]	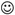	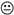	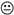	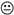	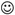	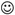	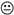
Saccardi et al. 2012 [26]	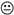	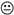	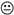	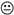	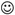	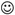	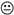
Vimercati et al. 2012 [27]	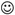	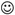	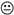	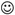	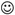	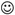	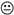
Mangler et al. 2013 [28]	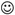	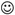	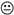	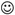	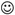	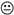	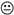
Kanté et al. 2017 [29]	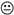	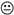	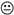	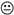	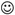	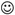	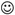
Alborzi et al. 2018 [30]	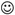	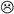	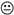	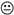	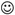	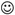	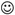
Guerriero et al. 2018 [31]	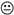	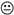	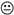	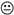	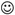	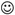	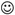

Notes: Legend: 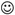: low risk; 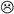: high risk; 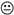: unclear risk.

**Table 2:** TVS versus MRI. Characteristics of studies included in the meta-analysis.

Authors and Year	Study Design	Index Test	Patients Treated (*n*)	Mean/Median Age	Mean BMI (kg/m^2^)	DPE Confirmed Localization (% (*n*))	Reference Standard
Abrao et al. 2007 [17]	cross-sectional study	TVS MRI	104	33.8 ± 6.1	NS	64.3 (63)	surgery
Bazot et al. 2009 [21]	retrospective study	TVS MRI RES	92	31.8 (20–51)	NS	97.8 (90)	histology
Saba et al. 2012 [23]	prospective study	TVS MRI	59	33 (21–44)	NS	76.3 (45)	surgery and histology
Cazalis et al. 2012 [24]	retrospective study	TVS MRI RES	25	35.4 ± 6.1	NS	100 (25)	surgery and histology
Fiaschetti et al. 2012 [25]	prospective study	TVS MRI	58	34 ± 6	NS	71.2 (57)	surgery
Saccardi et al. 2012 [26]	prospective study	TVS MRI	54	32.3 ± 5.8	20.6 ± 2.2	85.2 (46)	surgery and histology
Vimercati et al. 2012 [27]	prospective study	TVS MRI	90	34	NS	82.2 (74)	surgery and histology
Mangler et al. 2013 [28]	prospective study	TVS MRI RES	79	34 (19–51)	23 (17–35)	61.0 (48)	surgery and histology
Alborzi et al. 2018 [30]	prospective study	TVS MRI RES	317	31 ± 5.4	NS	79.5 (252)	surgery and histology
Guerriero et al. 2018 [31]	prospective study	TVS MRI	159	33 ± 7	nr	66.6 (106)	surgery

TVS: trans-vaginal sonography; MRI: magnetic resonance imaging; RES: rectal endoscopy sonography.

**Table 3:** **TVS versus RES.** Characteristics of studies included in the meta-analysis.

Authors and Year	Study Design	Test Method	Patients Treated (*n*)	Mean/Median Age	Mean BMI (kg/m^2^)	DPE Confirmed Localization (% (*n*))	Reference Standard
Bazot et al. 2003 [15]	prospective study	TVS RES	30	32 (21 ± 50)	NS	93.0 (28)	histology
Bazot et al. 2007a [18]	prospective study	TVS RES	81	31.9 (20–51)	NS	97.5 (79)	surgery and histology
Bazot et al. 2009 [21]	retrospective study.	TVS MRI RES	92	31.8 (20–51)	NS	97.8 (90)	histology
Bergamini et al. 2010 [22]	prospective study	TVS RES	61	33.1 (28–37)	NS	83.6 (51)	surgery and histology
Cazalis et al. 2012 [24]	retrospective study.	TVS MRI RES	25	35.4 ± 6.1	NS	100 (25)	surgery and histology
Mangler et al. 2013 [28]	prospective study	TVS MRI RES	79	34 (19–51)	23 (17–35)	61.0 (48)	surgery and histology
Piketty et al. 2009 [20]	prospective study	TVS RES	134	32.1 ± 5.0 (22–47)	NS	100 (134)	histology
Alborzi et al. 2018 [30]	prospective study	TVS MRI RES	317	31 ± 5.4	NS	79.5 (252)	surgery and histology

Notes: TVS: trans-vaginal sonography; MRI: magnetic resonance imaging; RES: rectal endoscopy sonography.

**Table 4:** MRI versus RES. Characteristics of studies included in the meta-analysis.

Authors and Year	Study Design	Index Test	Patients Treated (*n*)	Mean/Median Age	Mean BMI (kg/m^2^)	DPE Confirmed Localization (% (*n*))	Reference Standard
Bazot et al. 2007b [19]	prospective study	MRI RES	88	32.1 (20–51)	NS	97.7 (86)	surgery and histology
Bazot et al. 2009 [21]	retrospective study	TVS MRI RES	92	31.8 (20–51)	NS	97.8 (90)	surgery and histology
Cazalis et al. 2012 [24]	retrospective study	TVS MRI RES	25	35.4 ± 61	NS	100 (25)	surgery and histology
Chapron et al. 2004 [16]	retrospective study	MRI RES	81	31.9 ± 6.7	22.3 ± 3.3	100 (81)	histology
Kanté et al. 2017 [29]	retrospective study	MRI RES	239	33 (20–53)	22 (16–38)	NS	surgery and histology
Mangler et al. 2013 [28]	prospective study	TVS MRI RES	79	34 (19–51)	23 (17–35)	61.0 (48)	surgery and histology
Alborzi et al. 2018 [30]	prospective study	TVS MRI RES	317	31 ± 5.4	NS	79.5 (252)	surgery and histology

TVS: trans-vaginal sonography; MRI: magnetic resonance imaging; RES: rectal endoscopy sonography.

## Supplementary Materials

The following are available online at https://www.mdpi.com/2075-4418/9/4/225/s1, Figure S1: PRISMA 2009 Checklist; Table S1: DTA of TVS vs MRI in sites with few studies (univariate approach); Table S2: DTA of TVS vs RES in sites with few studies (univariate approach); Table S3: DTA of MRI vs RES in sites with few studies (univariate approach).
